# Examining the Relationship Between Depression, Rumination, and Anxiety: Insights from the DiSCoVeR Trial

**DOI:** 10.1192/j.eurpsy.2025.677

**Published:** 2025-08-26

**Authors:** L. Rubene, L. Konosonoka, A. Sturmane, E. Dechantsreiter, F. Padberg, D. Bavelier, F. Hummel, T. Cohen, N. Lerner, O. Bonne, Y. Benjamini, A. Lotan, M. Nahum, E. Rancans

**Affiliations:** 1 Department of Psychiatry and Narcology, Riga Stradiņš University; 2 Riga Centre of Psychiatry and Addiction Disorders, Riga, Latvia; 3 Department of Psychiatry and Psychotherapy, LMU University Hospital, Munich, Germany; 4 Campus Biotech & Faculty of Psychology and Educational Sciences, University of Geneva; 5 Neuro-X Institute (INX) and Brain Mind Institute (BMI), École Polytechnique Fédérale de Lausanne (EPFL), Geneva, Switzerland; 6 School of Occupational Therapy, Faculty of Medicine; 7 Department of Psychiatry, Hadassah Medical Center, Faculty of Medicine; 8 Department of Statistics and Data Science, The Hebrew University of Jerusalem; 9 Department of Adult Psychiatry and the Biological Psychiatry Lab, Hadassah University Medical Center, Jerusalem, Israel

## Abstract

**Introduction:**

The DiSCoVeR trial (The DiSCoVeR Project: Examining the synergistic effects of a cognitive control videogame and a self-administered non-invasive brain stimulation on alleviating depression) is a double-blind, sham controlled, randomized controlled trial (RCT) investigating the feasibility and efficacy of an innovative, self-applied treatment approach for patients diagnosed with major depressive disorder (MDD). The multi-site trial is conducted at three clinical trial sites (Hadassah, Israel; Riga Stradiņš University, Latvia; Ludwig-Maximilian-University, Germany). During the first study visit of this trial data on different patient baseline parameters were gathered including assesment of depressive symptoms, anxiety symptoms and rumination.

**Objectives:**

The aim of this abstract is to examine the relationship between depression, rumination and anxiety in this patient sample. Rumination, often characterized by repetitive, negative thinking, can exacerbate symptoms of anxiety and depression by maintaining and intensifying negative emotional states. This cycle creates a challenging clinical problem making it difficult to break free without targeted interventions.

**Methods:**

This analysis includes baseline data from 106 MDD patients enrolled in the DiSCoVeR trial as of April 2024. Depression severity was assessed using the Montgomery–Åsberg Depression Rating Scale (MADRS), anxiety symptoms were measured using the Generalized Anxiety Disorder Questionnaire (GAD-7), and rumination was evaluated with the Ruminative Response Scale (RRS). Data were analyzed using the Jamovi statistical platform, applying linear regression model to explore the relationship between depression, rumination, and anxiety. All assumptions for linear regression were met prior to analysis.

**Results:**

The mean age of the participants in this study sample ranged from 18 to 63 years old (mean age 33.4 years). 65.7% of the participants were female. Regression analysis revealed a significant positive association between anxiety (GAD-7) and rumination (RRS), suggesting that increased anxiety symptoms are associated with higher levels of rumination (p < .001). However, age and gender were not significant predictors of rumination. While depression (MADRS) was moderately associated with rumination, this effect was not statistically significant. Educational level showed a marginal effect, with university-educated individuals showing higher rumination levels compared to those with professional education.

**Conclusions:**

In this patient sample overall, anxiety (GAD-7 score) was the strongest predictor of rumination, while other factors such as depression, age, and gender did not show significant effects. Education level might have a marginal impact, especially for individuals with university education.

**Disclosure of Interest:**

None Declared

